# Better performance for right-skewed data using an alternative gamma model

**DOI:** 10.1186/s12874-023-02113-1

**Published:** 2023-12-15

**Authors:** Peter Veazie, Orna Intrator, Bruce Kinosian, Ciaran S. Phibbs

**Affiliations:** 1 Canandaigua Veterans Affairs Medical Center, 400 Fort Hill Ave., Canandaigua, New York 14424 USA; 2 Department of Public Health Sciences, University of Rochester Medical Center, 265 Crittenden Blvd. CU 420644, Rochester, NY 14642 USA; 3 Cpl Michael J. Crescenz Veterans Affairs Medical Center, 3900 Woodland Ave, Philadelphia, PA 19104 USA; 4 University of Pennsylvania, Philadelphia, PA 19104 USA; 5 Palo Alto Veterans Affairs Health Care System, 750 Willow Road (MPD 152), Menlo Park, CA 94025 USA; 6 Stanford University, 453 Quarry Road, MC 5660, Palo Alto, CA 94304 USA

**Keywords:** Gamma distribution, Generalized Linear models, Maximum likelihood estimation, Right-skewed variables

## Abstract

**Background:**

The Maximum Likelihood Estimator (MLE) for parameters of the gamma distribution is commonly used to estimate models of right-skewed variables such as costs, hospital length of stay, and appointment wait times in Economics and Healthcare research. The common specification for this estimator assumes the variance is proportional to the square of the mean, which underlies estimation and specification tests. We present a specification in which the variance is directly proportional to the mean.

**Methods:**

We used simulation experiments to investigate finite sample results, and we used United States Department of Veterans Affairs (VA) healthcare cost data as an empirical example comparing the fit and predictive ability of the models.

**Results:**

Simulation showed the MLE based on a correctly specified alternative has less parameter bias, lower standard errors, and less skewness in distribution than a misspecified standard model. The application to VA healthcare cost data showed the alternative specification can have better R square, smaller root mean squared error, and smaller mean residuals within deciles of predicted values.

**Conclusions:**

The alternative gamma specification can be a useful alternative to the standard specification for estimating models of right-skewed continuous variables.

**Supplementary Information:**

The online version contains supplementary material available at 10.1186/s12874-023-02113-1.

## Introduction

Estimating model parameters for right-skewed distributions, such as for costs, hospitalization events, and length of hospital stays are important to research on healthcare and health services, and more generally to research in economics and social sciences, among other fields. For example, statistical models of the impact of long-term services and supports on overall costs of care from a health system perspective, if misspecified, can provide poor predicted costs for older frail populations thereby impacting real-world budgeting and policy decisions. Since costs tend to be right skewed, they, and other right-skewed variables, are often modeled using a gamma distribution [1–7]. For example, Hong et al. used the gamma Generalized Linear Model (GLM) to analyse total and out-of-pocket health care expenditures from the Medical Organizations Survey linked to the Medical Expenditure Panel Survey to evaluate the impact of Accountable Care Organizations on adults in the United States [8]. Barnett et al. used the gamma GLM to evaluate determinants of healthcare cost among Veterans with HIV from United States Department of Veterans Affairs (VA) data [9]. Graves et al. used the gamma GLM to analyse the effect of respiratory track, urinary track, and hospital-acquired infections on both hospital length of stay and costs among adults in a prospective cohort study of two Australian hospitals [6]. Nikolovaa et al. used the gamma GLM to analyse, and compare to alternative models, appointment waiting times using the Scottish morbidity record [7].

Methods for predicting values of right-skewed variables and estimating characteristics of their distributions are continually being investigated: for example, Machine Learning and nonparametric techniques are being developed in this context [10–12]. Nonetheless, parametric methods remain a common and useful approach [13–15]. However, because the characteristics of right-skewed variables can vary across populations and type of outcome, no single technique or model is universally preferred. As Basu and Manning conclude in their review of cost modeling methods, “No current method is optimal or dominant for all cost applications” [16]. Rather than seek a methodological panacea, researchers can benefit from greater flexibility by expanding their toolbox and thereby increase the ability to select an approach appropriately aligned with their goals and data. In this paper, we recommend expanding the parametric modeling toolbox to include an alternative specification to the common gamma model.

Although use of the gamma distribution is common [1–9], this specification of such models is not always the best option. For example, using VA data, Wagner et al. [17] investigated the gamma distribution in their analysis of cost-based risk scores, and Gao et al. [18] investigated the gamma distribution in their development of a case-mix algorithm for hospitals and payers to compare their providers’ cost performance. Both studies found the gamma model performed poorly. In this paper we show that such results do not imply the gamma distribution is necessarily inappropriate for the data. Another specification for the gamma model may perform well.

Gamma distribution models of positive variables are commonly based on two parameters: a scale parameter and a shape parameter. The mean of the distribution is equal to their product. In these models, the conditional expectation is commonly specified with the influence of covariates through the scale parameter (the gamma scale model), and consequently the conditional variance is specified as being proportional to the square of the conditional mean. Both the Maximum Likelihood Estimator (MLE) and the Maximum Quasi-Likelihood Estimator (MQLE) tend to encode this specification. For example, see the Stata glm function [19] and the SAS statistical software’s proc glm procedure [20]. Specification tests for the gamma distribution also often take advantage of this moment condition: for example, see the Modified Parks Test [3, 21].

In this paper we focus on the gamma shape model, a model specification in which covariates influence the distribution through the shape parameter such that the variance is directly proportional to the mean [22, 23]. We show that the gamma shape model can be important for identifying a statistically adequate model using Monte Carlo simulation, and we show that, relative to the standard specification, it can provide better predictions, using data from the United States Department of Veterans Affairs.

## Methods

In this section, we present the gamma shape model and the methods we used for evaluating the estimator’s performance.

### Alternative gamma model specification

A random variable, *Y*, with a range on the positive real line, has a gamma distribution if its probability density function is.

$$f\left(y\right)=\frac1{\mathrm\Gamma\left(a\right)\cdot\beta^a}\cdot y^{a-1}\cdot e^{-\frac y\beta}$$

in which the shape parameter is denoted as α, and the scale parameter is denoted as β. In terms of these parameters, the mean of *Y* is the shape parameter multiplied by the scale parameter:

$$E\left(Y\right)=\alpha\cdot\beta$$

The variance of *Y* is the shape parameter multiplied by the square of the scale parameter:

1 $$V(Y)=\alpha \cdot {\beta ^2}$$

The standard gamma scale model takes advantage of the fact that, by multiplying and dividing the variance by the shape parameter *α*, the variance can be expressed as.

$$V\left(Y\right)=\frac1\alpha\cdot\left(\alpha\cdot\beta\right)^2$$

Because α⋅β is the mean, the variance is proportional to the mean squared:

2 $$V(Y)=\frac{1}{\alpha } \cdot E{(Y)^2}$$

However, by inspecting Eq. 1, the variance is also directly proportional to the mean. The variance can be expressed as.

$$V\left(Y\right)=\beta\cdot\left(a\cdot\beta\right)$$

which is

3 $$V(Y)=\beta \cdot E(Y)$$

The difference between Eqs. 2 and 3 is of little importance when considering a single distribution as they equate to the same value. However, the distinction can be important when considering conditional distributions across the range of covariate values. If distributions are nontrivially conditional on other variables, those variables must modify the parameters of the distribution. Consequently, in terms of the preceding gamma distribution, if predictors influence the mean, they must do so by influencing either the shape parameter or the scale parameter (or both). For example, if the distribution of costs among those who are 60 years old is different from the distribution of costs among those who are 65 years old, then, assuming both are gamma distributed, either the shape parameter or the scale parameter (or both) must be different across the two groups.

If covariates affect only the scale parameter, then the conditional mean can be expressed as.

$$E\left(Y\vert\mathbf X=\mathbf x\right)=\alpha\cdot\beta\left(\mathbf x\right)$$

in which the scale parameter, *β*, is a function of random variables **X**, and the shape parameter, *α*, is constant: the mean is proportional to the scale parameter across the range of **X**. In this case, because we hold the shape parameter constant, the conditional variance, as a function of covariates, is proportional to the conditional mean squared, which is the typical specification:

4 $$V(Y|{\mathbf{X}}={\mathbf{x}})=\frac{1}{\alpha } \cdot E{(Y|{\mathbf{X}}={\mathbf{x}})^2}$$

If, instead, variables affect the mean through the shape parameter, *α*(**x**), and the scale parameter *β* is constant, then the conditional mean is proportional to the shape parameter across the range of covariates **X**:

$$E\left(Y\vert\mathbf X=\mathbf x\right)=\alpha\left(\mathbf x\right)\cdot\beta$$

Therefore, the conditional variance, as a function of covariates, is directly proportional to the conditional mean across the range of covariates **X**:

5 $$V(Y|{\mathbf{X}}={\mathbf{x}})=\beta \cdot E(Y|{\mathbf{X}}={\mathbf{x}})$$

Note that Eq. 5 does not contradict the exponential family consequence for the gamma distribution that the variance is proportional to the mean squared: this relationship holds for any specific gamma distribution (and thereby for any specified set of covariate values) because Eq. 1 implies Eq. 2, as well as Eq. 3. Equations 4 and 5, however, become different specifications when they are treated as functions of covariates rather than as specific distributions.

The difference between these two specifications can influence the parameter and standard error estimators. Although the regression functions of both specifications have the same parametric form, if the regressors are included in the wrong parameter (either shape or scale parameter), then the wrong moment condition across covariates is established in the estimation of the conditional likelihood function (i.e. either Eq. 4 or Eq. 5). Consequently, the model coefficients and standard error estimates are inconsistent. To maximize the likelihood function, the MLE will find the parameters that balance the regression function and the mean-variance moment condition. The MLE will adjust the regression coefficients to account for the incorrect moment condition thereby generating an inconsistent estimator and inconsistent standard error estimator.

The MQLE used to estimate parameters of Generalized Linear Models does not generally address this concern. Li and Xiru [24], Chen et al. [25], and Yin and colleagues [26, 27], among others, show that the MQLE is consistent; however, these results assume the variance is correctly specified up to a scale parameter of a function of the mean [28], which is the issue of concern here. Although misspecification of the variance can be addressed using nonparametric quasi-likelihood methods [29], a parametric estimator may be preserved if the gamma shape specification is statistically adequate.

In this work, we focus on the common application of the gamma distribution to random variables defined on the positive real line. However, the implications of this work also apply to random variables with greatest lower bounds other than 0, i.e. with *L* any real number in the more general statement for the distribution:

$$f\left(y\right)=\frac1{\mathrm\Gamma\left(\alpha\right)\cdot\beta^\alpha}\cdot\left(y-L\right)^{\alpha-1}\cdot e^\frac{\left(L-y\right)}\beta$$

for all *y* > *L*.

### Simulation

The asymptotic properties of MLE are known to depend on correct specification [30]; consequently, if a model is misspecified, whether as the gamma scale or gamma shape model, then MLE may not perform well. We used Monte Carlo experiments to provide an example of the finite sample properties of the properly specified gamma shape model and the consequence of using the misspecified gamma scale model in this case. The purpose of these results is not to prove that proper specification of a likelihood function is required for asymptotic efficiency and consistent estimation, which is well established in the MLE literature [30], nor are results intended to prove that the models are always importantly different, as that depends on the data. We present results as an example to show that specification *can* meaningfully matter and thereby support the claim that the gamma shape model should be considered when modeling positive right skewed data, particularly if the gamma scale model is not fitting well.

For the purpose of this investigation, we used the common log-link function for the GLM, i.e. we specify the mean as the natural exponential of predictors:

$$E(Y|{\mathbf{X}}={\mathbf{x}})={e^{\theta ^{\prime}{\mathbf{x}}}},$$

in which θ denotes a vector of coefficients. We used 10,000 Monte Carlo samples, with sample sizes of 1000 observations each, to compare the scale and shape models. The Monte Carlo samples were drawn from a gamma distribution with a uniform distributed predictor variable on the interval of 0 to 2, denoted below as *x*, influencing the shape parameter, and with the scale parameter specified as a constant:

$$\alpha\left(x\right)=e^{2\cdot x+0.05\cdot x^2+1}$$

and

$$\beta ={e^3}.$$

We estimated both the gamma scale and gamma shape specifications with MLE on each data set, and we compared the averages and standard deviations of the coefficient estimates, the average standard error estimates, and the skewness and kurtosis of the estimate distributions.

### Modeling costs of healthcare in the U.S. department of veterans affairs

We examined an adaptation of a Medicare Advantage capitation index (the CMS V21 risk score) based on Hierarchical Condition Categories (HCC), which is a projection to a cost index used to capitate Medicare payments to Medicare Managed Care organizations [31]. Acknowledging the under-reporting of diagnoses in VA data and its negative impact on the use of the CMS V21 risk score in the VA, Wagner et al. [17] created the Nosos risk score. The Nosos is a risk adjusted cost model that uses the V21 risk score and adds 48 mental health diagnoses relevant to the cost of VA care and 24 drug categories, as well as age, age squared, indicators of being white, being male, having insurance, being married, being on a VA chronic illness registry, and priority status group indicators (which indicates levels of service-related disability). We further modified the Nosos by including the individual indicators of HCC’s that comprise the V21 risk score rather than incorporating the V21 score itself.

We used data from VA inpatient, outpatient, and fee-basis files, cleaned Managerial Cost Accounting costs, enrollment and vital status for fiscal year 2017. Medicare inpatient, Carrier and certain outpatient claims supplemented VA diagnoses. The dependent variable was the total outlier corrected Consumer Price Index adjusted VA cost. We applied the cost models to five populations of Veterans with varying frailty levels: [1] all Veterans using VHA; [2] all Veterans using any one of the VA Geriatrics & Extended Care services (GEC Cohort); [3] all Veterans using VA’s Home-Based Primary Care, a program that provides interdisciplinary care (physicians and nurse practitioners, nurses, social workers, therapists, dieticians, clinical pharmacists, and other professional care) to Veterans who are unable to leave their homes to receive care in clinics at their homes; [4] all Veterans who used VA services with JEN Frailty Index (JFI) less than six (corresponding to having no more than 1 Activity of Daily Living (ADL) impairment); and [5] those with JFI between six and twelve, corresponding to having two or more ADL impairments [32].

We estimated both the gamma shape and scale models as well as the more complex gamma shape/scale model in which covariates influence both parameters. We used MLE in the Stata statistical software version 17. See the Appendix for the Stata code to estimate the gamma shape model. To evaluate the within-sample predictive ability of each model, we used the R square to compare prediction (calculated as 1 minus the ratio of the residual variance to the total variance), the square-root of the mean squared error to compare model precision, and the maximum average residual across deciles of predicted values to compare maximum residual deviation.

## Results

### Simulation results

Table 1 presents the average across 10,000 Monte Carlo samples of the coefficient estimates, the standard deviations of the estimates, the average of the standard error estimates, and the skewness and kurtosis of estimates for each model and parameter. The correctly specified gamma shape model has biases in the data of 0%, 2%, and 0.2% for the coefficients on *x* and *x* ^2^, and the constant, respectively. Whereas, the misspecified gamma scale model has larger biases of 2%, 28%, and 2% for the coefficients on *x* and *x* ^2^, and the constant, respectively. More striking, however, is the standard deviation of the estimates for the gamma shape model is approximately half of the corresponding standard deviations for the gamma shape model for each coefficient. Moreover, the mean estimated standard errors are the same as the standard deviations for the gamma shape model, but they are 26%, 14%, and 40% lower than the standard deviations in the gamma scale model for the coefficients on *x* and *x* ^2^, and the constant, respectively. Regarding the distribution of the coefficient estimates, there is no evidence that the gamma shape model estimates deviate from the Normal distribution in skewness and kurtosis; however, for the gamma scale model, estimates deviate from Normal in terms of skewness (p values < 0.000 for each coefficient), which can explain why its estimated standard errors do not match the standard deviations.

**Table 1:** Monte Carlo simulation results for the distribution of estimates from 10,000 iterations for the gamma scale (incorrect specification) and gamma shape (correct specification) models

Coefficient	Model	Mean	Standard Deviation	Mean Standard Error	Skewness (p value)	Kurtosis (p value)	Joint test *p* value^a^
${\theta _x}$= 2	Scale	2.037	0.393	0.290	0.145 (0.000)	3.064 (0.189)	0.000
	Shape	2.000	0.166	0.166	-0.016 (0.505)	2.935 (0.182)	0.328
${\theta _{{x^2}}}$= 0.05	Scale	0.036	0.163	0.141	-0.120 (0.000)	3.053 (0.272)	0.000
	Shape	0.051	0.069	0.069	0.035 (0.151)	2.926 (0.124)	0.110
${\theta _{Const}}$= 1	Scale	0.978	0.210	0.126	-0.158 (0.000)	3.059 (0.228)	0.000
	Shape	0.998	0.099	0.099	-0.032 (0.188)	3.059 (0.225)	0.202

^a^Joint test of skewness = 0 and kurtosis = 3

### Healthcare costs in the U.S. department veteran affairs results

Table 2 presents the R square, root means squared error, and maximum mean decile errors for the two models on each of the five populations of U.S. Veterans. The R square was larger for the gamma shape model than the gamma scale model across all populations. Indeed, the R squares are negative for the gamma scale model estimated on the overall non-institutional population and population with JFI less than six, whereas the gamma shape model has R squares of 0.57 and 0.41 in these populations, respectively. The negative R squares indicate that the estimated regression function strongly deviates from the underlying true conditional expectation. The root mean squared error was 15–85% smaller for the gamma shape model across all populations. In addition, the maximum mean error across deciles of predicted values was smaller by approximately one order of magnitude for the gamma shape model in all populations. In contrast, for this data, when allowing covariates to impact both shape and scale parameters, results fell between the gamma shape and gamma scale model results on each measure.

**Table 2:** Prediction criteria results of the gamma scale and gamma shape and gamma shape/scale models for noninstitutionalized veterans in fiscal year 2017

		Population				
**Criterion**	**Model** ^a^	**Overall**	**GEC Cohort**	**HBPC**	**JFI < 6**	**JFI ≥ 6**
N		4 838 736	330 358	69 649	4 427 618	411 118
R^2^	Scale	Neg^b^	0.40	0.45	Neg^b^	0.37
	Shape	0.57	0.62	0.61	0.41	0.57
	Both	Neg^b^	0.51	0.50	Neg^b^	0.45
RMSE	Scale	118 526	52 099	44 439	25 483	55 478
	Shape	18 592	41 585	37 501	11 973	45 826
	Both	108 444	46 881	42 301	22 458	51 790
Max Error	Scale	-56 164	-46 181	-24 676	-17 232	-46 239
	Shape	8 369	9 198	9 789	4 675	9 436
	Both	-49 436	-27 233	-14 737	-13 753	-32 706

^a^Scale = gamma scale model; Shape = gamma shape model; Both = gamma shape/scale model with covariates impacting both shape and scale parameters

^b^Neg denotes negative R square values, which is possible if the regression model does not match the true conditional expectations

## Discussion

Simulation results showed what we expected, given the requirements for efficiency and consistent estimation: The correctly specified model, in this case the gamma shape specification, showed less bias in the data (up to 93% lower percent bias on estimated coefficients in this example), which can affect the accuracy of predicted values. Notably, however, the correctly specified model can also have smaller standard deviations of the coefficient sampling distributions (up to 58% lower in this example) and more accurate estimated standard errors (the standard deviations and estimated standard errors were the same for the correct specification but up to 40% off for the misspecified model). Moreover, the correct specification had less skewness in the distribution of estimates (up to 89% lower magnitude in skewness in this example). These results indicate the potential for improved statistical inferences and more appropriate use of standard test statistics based on the normal distribution. The larger R square, smaller root means squared error, and smaller maximum mean decile errors in the empirical example provides evidence that the gamma shape model can have better predictive ability than the gamma scale specification in real-world data—in some cases, considerably better. Because the difference between R square values across two models estimated on the same population using the same covariates is proportional to the difference in the variation of the model bias across the range of covariates, these results show that the gamma shape model had considerably lower model bias than the typical gamma scale model for this data. Although not a general replacement for the gamma scale model, which would outperform the gamma shape specification if it better matched the underlying data generating process, these results strongly suggest researchers consider the gamma shape specification among the set of models they use to model right-skewed variables.

An additional implication of the alternative specification is regarding the interpretation of the commonly used Modified Park’s Test (MPT) for specification of the GLM family [3, 21]. The MPT tests whether the variance is proportional to the square of the mean across the range of covariates. If the hypothesis is rejected, then the MPT is taken to imply the distribution is not a gamma. Clearly, this need not be true. Under the gamma shape specification, the variance is directly proportional to the mean across the range of covariates. Rejecting the moment condition of the gamma scale specification leaves open the possibility that the distribution remains a gamma, but covariates influence the distribution through the shape parameter. Moreover, if we reject both moment conditions, this still does not imply the distribution is not gamma because if predictors influence the mean through both the shape and scale parameters, then a consistent moment condition does not hold across the range of covariate values and the Modified Park test simply does not apply.

To identify misspecification and differentiate the gamma shape and gamma scale models, one can use a model fit statistic such as the Veazie-Ye goodness-of-fit test [33] for each specification, which tests the gamma distribution with the specified parameterization. Or, one can estimate the gamma shape/scale model and use a joint test of each coefficient vector to determine which is affected by the variables; this assumes the gamma distribution but tests parameterization (see Appendix for estimation and testing code for use with the Stata statistical software program).

This paper does not present a survey of all possible gamma specifications. However, if neither the scale or shape models are statistically adequate, other parameterizations can be considered as well. See Venter [23] for examples in which the gamma can be specified such that its variance can be expressed as any power of the mean. Moreover, if regressors affect both the scale and shape parameters, then the regression function can be based on the model in which regressors affect both parameters as mentioned in the preceding paragraph. However, due to greater complexity of the gamma shape/scale model, which has twice the number of parameters associated with the same number of covariates, estimation can be problematic for small samples and computationally challenging for larger samples.

## Conclusion

Parametric modeling is common for estimation, risk adjustment, and the identification of predictors of right-skewed outcomes. In this paper, we presented an alternative to an otherwise common gamma specification and showed that it can have better empirical performance. We recommend the alternative gamma shape model be added to the toolkit for modeling right-skewed continuous conditional distributions.

### Supplementary Information

**Additional file 1.**

## Data Availability

VA data are not publicly available.

## References

[1] Manning WG (1998). The logged dependent variable, heterscedasticity, and the retransformation problem. J Health Econ.

[2] Manning WG, Basu A, Mullahy J (2005). Generalized modeling approaches to risk adjustment of skewed outcomes data. J Health Econ.

[3] Manning WG, Mullahy J (2001). Estimating log models: to transform or not to transform?. J Health Econ.

[4] Griswold M, Parmigiani G, Potosky A, Lipscomb J (2004). Analyzing health care costs: Acomparison of statistical methods motivated by Medicare Colorectal cancer charges. Biostatistics.

[5] Mullahy J (2009). Econometric modeling of health care costs and expenditures: a survey of analytical issues and related policy considerations. Med Care.

[6] Graves N, Weinhold D, Tong E, Birrell F, Doidge S, Ramritu P (2007). Effect of healthcare-acquired Infection on length of hospital stay and cost. Infect Control Hosp Epidemiol.

[7] Nikolovaa S, Sinko A, Sutton M (2015). Do maximum waiting times guarantees change clinical priorities for elective treatment? Evidence from Scotland. J Health Econ.

[8] Hong YR, Sonawane K, Larson S, Mainous AG, Marlow NM (2018). Impact of provider participation in ACO Programs on Preventive Care Services, patient experiences, and Health Care expenditures in US adults aged 18–64. Med Care.

[9] Barnett PG, Chow A, Joyce VR, Bayoumi AM, Griffin SC, Nosyk B (2011). Determinants of the cost of health services used by veterans with HIV. Med Care.

[10] Mazumdar M, Lin JYJ, Zhang W, Li LH, Liu M, Dharmarajan K (2020). Comparison of statistical and machine learning models for healthcare cost data: a simulation study motivated by Oncology Care Model (OCM) data. BMC Health Serv Res.

[11] Duncan I, Loginov M, Ludkovski M (2016). Testing Alternative Regression frameworks for Predictive modeling of Health Care costs. N Am Actuar J.

[12] Morid MA, Kawamoto K, Ault T, Dorius J, Abdelrahman S (2017). Supervised Learning Methods for Predicting Healthcare Costs: Systematic Literature Review and Empirical Evaluation. AMIA Annu Symp Proc.

[13] Mihaylova B, Briggs A, O’Hagan A, Thompson SG (2011). Review of statistical methods for Analysing Healthcare Resources and costs. Health Econ.

[14] Kurz CF (2017). Tweedie distributions for fitting semicontinuous health care utilization cost data. Bmc Med Res Methodol.

[15] Jones AM, Lomas J, Rice N (2014). Applying Beta-type size distributions to Healthcare cost regressions. J Appl Economet.

[16] Basu A, Manning WG (2009). Issues for the Next Generation of Health Care cost analyses. Med Care.

[17] Wagner TH, Upadhyay A, Cowgill E, Stefos T, Moran E, Asch SM (2016). Risk Adjustment Tools for Learning Health systems: a comparison of DxCG and CMS-HCC V21. Health Serv Res.

[18] Gao J, Moran E, Almenoff PL (2018). Case-Mix for Performance Management: a risk Algorithm based on ICD-10-CM. Med Care.

[19] StataCorp (2023). Stata 18 Base Reference Manual.

[20] SAS Institute Inc (2013). SAS/STAT 13.1 user’s guide.

[21] Deb P, Norton EC (2018). Modeling Health Care expenditures and Use. Annu Rev Public Health.

[22] Corrales Bossio M, Cepeda Cuervo E (2015). Gamma regression models with the Gammareg R package. Comun en Estadistica.

[23] Venter GG (2007). Generalized linear models beyond the exponential family with loss reserve applications. Astin Bull.

[24] Yue L, Chen X (2004). Rate of strong consistency of quasi maximum likelihood estimate in generalized linear models. Sci China Ser Math.

[25] Chen KN, Hu IC, Ying ZL (1999). Strong consistency of maximum quasi-likelihood estimators in generalized linear models with fixed and adaptive designs. Ann Stat.

[26] Yin CM, Zhao LC, Wei CD (2006). Asymptotic normality and strong consistency of maximum quasi-likelihood estimates in generalized linear models. Sci China Ser A.

[27] Yin CM, Zhao LC (2005). Strong consistency of maximum quasi-likelihood estimates in generalized linear models. Sci China Ser A.

[28] Wedderburn RWM (1974). Quasi-Likelihood Functions, Generalized Linear-Models, and Gauss-Newton Method. Biometrika.

[29] Chiou JM, Muller HG (1999). Nonparametric quasi-likelihood. Ann Stat.

[30] Amemiya T (1985). Advanced Econometrics.

[31] Pope GC, Kautter J, Ellis RP, Ash AS, Ayanian JZ, Lezzoni LI (2004). Risk adjustment of Medicare capitation payments using the CMS-HCC model. Health Care Financ Rev.

[32] Kinosian B, Wieland D, Gu X, Stallard E, Phibbs CS, Intrator O (2018). Validation of the JEN frailty index in the National Long-Term Care Survey community population: identifying functionally impaired older adults from claims data. BMC Health Serv Res.

[33] Veazie P, Ye Z (2020). A simple goodness-of-fit test for continuous conditional distributions. Ratio Mathematica.

