# Supplementary material for: Better performance for right-skewed data using an alternative gamma model
Source: BMC Med Res Methodol. 2023 Dec 15;23:298. doi: 10.1186/s12874-023-02113-1 (PMC10722755; doi:10.1186/s12874-023-02113-1)
Supplement: Supplementary file 1 — Additional file 1. [file 12874_2023_2113_MOESM1_ESM.docx]

**Appendix**

# Stata code for estimation

The following Stata code provides the MLE estimator of the Gamma shape model specification with a logarithm link function using with the ml suite of commands.

program mlfgamma_shape

version 17

args lnf xmean xbeta

quietly {

tempvar m a b

g double `m' = exp(`xmean')

g double `b' = exp(`xbeta')

g double `a' =`m'/`b'

replace `lnf' = ln(gammaden(`a', `b', 0, $ML_y1))

} //endquietly

end

The following Stata ml commands are then used for estimation:

ml model lf mlfgamma_shape (mean: DEPENDENT VARIABLE = LIST OF INDEPENDENT VARIABLES) (Scale:)

ml max

In these commands, the phrase “DEPENDENT VARIABLE” is a place holder for the variable name denoting the outcome variable, and the phrase “LIST OF INDEPENDENT VARIABLES” is a place holder for the list of variable names denoting the covariates. By copying the program and the ml commands into a Stata do file, the alternative specification can be estimated. Note that no covariates are listed in the scale parameter, which remains a constant in the Gamma shape model. However, the following code allows for directly estimating coefficients on variables directly affecting both the shape and scale parameters rather than estimating the mean and scale parameters. This specification allows for testing which of the shape or scale parameters are influenced by covariates and can also be used as a general specification that allows covariates to influence the mean through both shape and scale parameters.

program mlfgamma

version 17

args lnf xshape xscale

quietly {

tempvar a b

g double `a' = exp(`xshape')

g double `b' = exp(`xscale')

replace `lnf' = ln(gammaden(`a', `b', 0, $ML_y1))

} //endquietly

end

The following commands are used to estimate the preceding model and test whether covariates have significant effects within each of the shape and scale parameters:

ml model lf mlfgamma (Shape: DEPENDENT VARIABLE = LIST OF INDEPENDENT VARIABLES) ///

(Scale: LIST OF INDEPENDENT VARIABLES)

ml max

test [Shape]

test [Scale]

The test commands return joint tests that all variable coefficients are equal to 0 in each of the Shape and Scale equations. If the joint test of coefficients in an equation is strongly not significant, then there is little evidence that the variables influence the corresponding parameter. If both are significant, then the results from the preceding mlfgamma program can be used directly as a more general model. However, the mlfgamma program provides the relationship between variables and the shape and scale parameters rather than directly with the mean; consequently, if using the log-link function, coefficients must be added across each parameter equation for the same covariates to obtain the effect of the covariate on the mean. Although the association with the mean can be derived from the parameter-specific estimates using the mlfgamma program, one could simply include variables in the Scale component of the ml command associated with the mlfgamma_shape program above, thereby allowing the scale parameter to also be a function of variables and the mean function directly estimated. For example, using the mlfgamma_shape program the ml command would be written as

ml model lf mlfgamma_shape (mean: DEPENDENT VARIABLE = LIST OF INDEPENDENT VARIABLES) ///

(Scale: LIST OF INDEPENDENT VARIABLES)

ml max

Both the mlfgamma_shape and the mlfgamma programs can therefore allow for variables to influence both parameters and thereby provide a more general model than the Gamma shape and Gamma scale models compared herein. It should be noted that the list of variables included in each component of the models do not have to be the same.
